# Supplementary material for: Depression in association with neutrophil-to-lymphocyte, platelet-to-lymphocyte, and advanced lung cancer inflammation index biomarkers predicting lung cancer survival
Source: PLoS One. 2023 Feb 24;18(2):e0282206. doi: 10.1371/journal.pone.0282206 (PMC9956881; doi:10.1371/journal.pone.0282206)
Supplement: S1 Table — (PDF) [file pone.0282206.s001.pdf]

Table S1. Overall p-values from univariate linear analyses.

Variable	NLR	PLR	ALI
ECOG (0, 1, 2 or more)	0.28	0.17	0.13
Age	0.15	0.06	0.30
Race (white, non-white)	0.04	0.89	0.09
Gender	0.75	0.65	0.88
BMI	0.01	0.03	<0.001[1]
Education Level (high school or less, beyond high school)	0.09	0.67	0.08
Smoking Status	0.93	0.91	0.61
Marital Status (partnered/married, not married)	0.24	0.67	0.56
Cell Type	0.67	0.50	0.87
Treatment type	0.29	0.53	0.14

Note: outcomes were log-transformed due to right-skewed data distribution.
[1] BMI not included in ALI regression; BMI is an element in ALI calculation.
