# Supplementary material for: Depression in association with neutrophil-to-lymphocyte, platelet-to-lymphocyte, and advanced lung cancer inflammation index biomarkers predicting lung cancer survival
Source: PLoS One. 2023 Feb 24;18(2):e0282206. doi: 10.1371/journal.pone.0282206 (PMC9956881; doi:10.1371/journal.pone.0282206)
Supplement: S2 Table — (PDF) [file pone.0282206.s002.pdf]

Table S2. Overall p-values from univariate Cox regression.

Variable	P-value
ECOG (0-1, 2 or more)	0.04
Age (younger than 65, 65 or older)	0.02
Race (white, non-white)	0.80
Gender	0.35
BMI	0.33
Education Level (high school or less, beyond high school)	0.11
Smoking Status	0.21
Marital Status (partnered/married, not married)	0.27
Cell Type	0.88
Treatment type	0.29
